# Detection of *mecA* positive staphylococcal species in a wastewater treatment plant in South Africa

**DOI:** 10.1007/s11356-023-30319-9

**Published:** 2023-10-21

**Authors:** Adegboyega Oyedele Oladipo, Oluwatosin Gbemisola Oladipo, Carlos Cornelius Bezuidenhout

**Affiliations:** 1 Unit for Environmental Sciences and Management, Microbiology Group, North-West University, Potchefstroom Campus, Private Bag X6001, Potchefstroom, 2520 South Africa; 2 Department of Medical Microbiology and Parasitology, Obafemi Awolowo University Teaching Hospitals Complex, Ile-Ife, Nigeria; 3 Department of Microbiology and Biotechnology, Faculty of Natural and Applied Sciences, First Technical University, Ibadan, Nigeria

**Keywords:** Antibiotic resistance genes, Coagulase negative Staphylococci, *mecA* gene, Methicillin-resistant *Staphylococcus aureus*, Protein A gene (*spa*), Wastewater treatment plant

## Abstract

We investigated the prevalence of antibiotic resistant staphylococci and detection of resistant, virulence, and *Spa* genes in a South African wastewater treatment plant. Species identified were *Staphylococcus aureus*, *S. lentus*, *S. arlettae*, *S. cohnii*, *S. haemolyticus*, *S. nepalensis*, *S. sciuri *(now *Mammaliicoccus sciuri*), and *S. xylosus*. Isolates showed high resistance to methicillin (91%), ampicillin (89%), ciprofloxacin (86%), amoxycillin (80%), ceftazidime (74%), and cloxacillin (71%). Multiple antibiotic resistance (MAR) index for the isolates exceeded 0.2 (0.50–0.70). Among the isolates, 77% were *mecA*-positive. All *S. aureus* strains were positive for *nuc* and 7 *Spa* gene types. The present study highlights possibility of treated wastewaters being potential reservoir for antibiotic-resistant staphylococci. This is a cause for concern as wastewater effluents are decanted into environmental waters and these are, in many cases, used for various purposes including recreation (full contact), religious (full body submersion), and drinking water for some rural communities and water for livestock.

## Introduction

Due to increasing human population and urbanization as well as changing climatic conditions, the challenge of water scarcity has heightened in many developed and developing countries (du Plessis 2019a). South Africa is no exception, and extended droughts in the catchment areas of reservoir dams in the Western and Eastern Cape, KwaZulu Natal, and Northern Cape were responsible for major metropolitan cities to implement water restrictions (Botai et al. 2019; du Plessis 2019b). Alternative water sources, such as the reclamation of wastewater treatment plant (WWTP) effluent for various purposes, become crucial (Salgot and Folch 2018).

Treated wastewater effluents are usually discharged into receiving waters and reused for various purposes such as preparing drinking water, agricultural irrigation and livestock water, recreation, and industrial purposes (Angelakis et al. 2018). Effectively treated wastewaters are determined by their quality which are dependent on the physico-chemical properties (pH, temperature, electrical conductivity, various chemical constituents such as phosphates, nitrogen containing compounds, and organic load metals) and microbial indicators of fecal contamination, mostly *E. coli* (Jordaan & Bezuidenhout 2013). Indiscriminate discharge of poorly treated or untreated wastewater effluents are major contributors to surface water pollution (Malassa et al. 2013; Amirsoleimani et al. 2019; Kiliça et al. 2023).

According to Börjesson et al. (2010), Said et al. (2017) and Azuma et al. (2022) wastewaters are potential sources for the dissemination of antibiotic resistant bacteria (ARB) such as staphylococcal species into natural water environments. These staphylococcal species rank high among the bacteria causing diseases. In addition, they have been incriminated for many human infections such as skin and soft tissue infections, surgical site/wound infections, pneumonia, septicemia, and bone infections (Nanoukona et al. 2017; Oladipo et al. 2019). Several studies have detected staphylococcal species in wastewaters (Börjesson et al. 2009; Goldstein et al. 2012; Gómez et al. 2016). Porrero et al. (2016) reported the presence of *Staphylococcus aureus* in WWTP in Madrid, Spain, while Faria et al. (2009) and Čuvalova et al. (2015) reported the survival of coagulase negative staphylococci (CoNS) in treated effluents and drinking water from Portugal and Slovak Republic, respectively. Specifically, Gómez et al. (2016) and Said et al. (2017) detected 5 coagulase negative staphylococci (CoNS) — *S. lentus*, *S. cohnii*, *S. sciuri*, *S. haemolyticus*, and *S. xylosus* in wastewaters in Spain and Tunisia. Borjesson et al. (2009) also identified *S. lentus*, *S. sciuri*, *S. cohnii*, and *S. haemolyticus* in a municipal wastewater treatment plant in Sweden.

*Staphylococcus aureus* may be associated with severe infection, hence the need to distinguish it from the opportunistic coagulase negative staphylococci. In routine laboratory practice, the production of coagulase is frequently used as the sole criterion to distinguish *S. aureus* from other staphylococci. The coagulase test is therefore an important distinguishing characteristic of staphylococci (Cheesbrough 2006). Those that are coagulase positive are generally regarded as *S. aureus* and are potential pathogens that are flagged for further diagnostic tests (Cheesbrough 2006), while the CoNS are generally regarded as non-pathogenic and are routinely disregarded in the clinical diagnostic sphere (Okwara et al. 2004).

Staphylococcal species may exhibit resistance towards beta-lactam antibiotics such as ampicillin, methicillin, and penicillin (Porrero et al. 2016; Thompson et al. 2012; Said et al. 2017). The World Health Organization (WHO 2017a) reported that in Africa, 80% of *Staphylococcus aureus* infections are methicillin resistant. Multi-drug antibiotic resistance traits and antibiotic resistance genes (ARGs) such as in MRSA and other CoNS species had been isolated from wastewaters (Börjesson et al. 2009; Thompson et al. 2012; Wan and Chou 2014; Boopathy 2017; Said et al. 2017). In Nigeria, more recent studies by Adekanmbi et al. (2019), Oladipo et al. (2019), and Adesoji et al. (2020) have also confirmed the presence of multi-drug antibiotic-resistant staphylococci and *mecA* gene from wastewater sources.

The confirmation of the presence of the *mecA* gene has been the “golden standard” for detection of methicillin-resistant *S. aureus* (MRSA) worldwide (Yang et al. 2009; Igbinosa et al. 2016). The *nuc* gene detection is a confirmatory test for *S. aureus* strains, while *pvl* is generally used as a marker for community acquired MRSA (Gillen et al. 2015). The *PVL* gene is a virulence factor, which can enhance the ability of the bacterium to cause severe infections in human and animal hosts. *Spa*-typing of *S. aureus* strains is an investigation which could provide useful insight and information into the virulence potentials and nature of *S. aureus* specie. This test may further assist in the grouping of isolates into clonal lineages and *S. aureus* populations (Kolawole et al. 2013).

Occurrence of MRSA and genes in wastewater effluents discharged into water environments has therefore raised public health concerns due to likely threats posed to the human communities which could lead to community acquired MRSA (CA-MRSA) infections (Börjesson et al. 2009, 2010; Plano et al. 2011; Rosenberg et al. 2012). In South Africa, there is limited data on the detection of staphylococcal species in WWTPs and whether these make it into receiving water bodies as well as their persistence in these waters (Chidamba et al. 2016).

The aim of this study was to determine (i) the prevalence of staphylococcal species that are resistant to methicillin and other related antibiotics and (ii) the presence of *mecA*, *nuc*, and *luk-pvl* genes and *spa* types in the resistant staphylococci isolated from a South African wastewater treatment plant using standard protocols.

## Materials and methods

### Description and treatment processes at the wastewater treatment plant sampled

Water samples were collected from a wastewater treatment plant (WWTP) in the North-West Province of South Africa. From this plant, four sites were sampled; these were influent, primary effluent, secondary effluent, and final effluent. The plant is a full scale-wastewater treatment plant which has a designed capacity of 45,000 m^3^ per day with the potential of receiving wastewater streams from domestic, industrial, agricultural, abattoirs, hospital, and storm water sources. The average flow to the works is 29,000 m^3^ per day. The influent receives raw sewage into the plant for treatment. The treatment plan employed at the WWTP for each of the four sites is influent — here, preliminary filtration/mechanical methods are used. For the secondary effluent, biological treatment option is utilized, while the final or tertiary effluent is the final stage of treatment where chemical treatment by chlorination is employed. Generally, chlorination at 5 mg/L is used to reduce *E. coli* levels to 0 cfu/100 ml and to reduce odor caused by microorganisms before discharge into receiving waters.

### Sampling description

Samples were collected from each of the four sites in sterile 500 mL Schott glass bottles each. Grab sampling technique was used, and triplicate samples were collected from each of the four sampling points weekly for a period of four months. The wastewater samples were then transported in ice chested coolers and preserved under refrigeration conditions for microbiological analyses. The latter were conducted within 12 h of collection.

### Microbiological analysis of wastewater samples and preliminary identification of staphylococcal species

About 100 mL of water samples collected were filtered using sterile 0.45 µm membrane filters. These filters were afterwards enriched in Bacto tryptic soy broth (soybean-casein digest medium; Becton Dickinson, USA) and were later placed onto Mannitol salt agar (Biotec Laboratories, Kentford, UK). The resulting yellow colonies were presumed to be *S. aureus.* These were afterwards confirmed by culturing on MRSA CHROMagar base (CHROMagar™ MRSA-ITK Diagnostics BV, Uithoorn, The Netherlands). The putative MRSA produced characteristic purple color on the chromogenic agar. Gram staining was used to ensure that isolates were Gram-positive cocci and have characteristic clusters. *Staphylococcus aureus* and other staphylococcal species were later confirmed by the coagulase and catalase tests using standard protocols (Cheesbrough 2006; Igbinosa et al. 2016). All isolated and identified staphylococci were subjected to antibiotic susceptibility testing using 13 antibiotics and the standard Kirby-Bauer’s disk diffusion technique (CLSI 2014).

### 16S rRNA gene–based identification of staphylococcal isolates

The Nucleospin® tissue extraction kit (Macherey–Nagel, Düren, Germany) was used, according to the manufacturer’s manual, to isolate genomic DNA. Briefly, 2 mL overnight broth cultures were centrifuged at 8000 rpm for 5 min at room temperature to harvest the cells. The supernatant was discarded, and pelleted cells were then resuspended in 100 µL T1 buffer. Quality and integrity of extracted DNA products were verified by micro-spectrophotometry and gel electrophoresis as described by Oladipo et al. (2018a).

### PCR amplification

The C1000TM thermal cycler (Bio-Rad, Hercules, CA, USA) was used to perform PCR reactions. 16S rRNA gene amplification was conducted using primer sets and PCR conditions detailed in Table 1. Each PCR reaction included positive and negative controls as described by Oladipo et al. (2018b).

**Table 1:** Primers used for the identification of staphylococcal species and the detection of marker genes

Primers	Primer sequence (5′–3′)	PCR Conditions	Size (bp)	References
27F 1492R	5′GAGTTTGATCATGGCTCAG3 5′GGTTACCTTGTTACGACTT3′	1 cycle of 2 min at 95 °C; 35 cycles of 30 s at 94 °C; 30 s at 53 °C for, 1 min at 72 °C; 1 cycle 10 min at 72 °C	1500	Lane (1991)
*Spa* 1095F new spa extend: f	5′-AGACGATCCWTCAGTGAGC-3′ 5′-TAATCCACCAAATACAGTTGTACC-3′	1 cycle of 5 min at 94 °C; 35 cycles of 45 s at 94 °C; 45 s at 62; 90 s at 72 °C, 10 min at 72 °C	200	Shopsin (1999)
*mec*A-F *mec*A-R	5′AACGATTGTGACACGATAGCC3′ 5′GGGATCATAGCGTCATTATC3′	1 cycle of 5 min at 94 °C; 35 cycles of 30 s at 94 °C; 30 s at 55 °C; 1 min at 72 °C	527	Kumar et al. (2016)
*nuc*-1 *nuc*-2	5′TCAGCAAATGCATCACAAACAG3′ 5′CGTAAATGCACTTGCTTCAGG3′	1 cycle of 5 min at 94 °C; 35 cycles of 30 s at 94 °C; 30 s at 55 °C; 1 min at 72 °C	255	Othman et al. (2014)
*luk*-F *luk*-R	5′ATCATTAGGTAAATGTCTGGCA TGATCC3′ 5′AGCATCAAGTGTATTGGATAGC AAAAGC3′	1 cycle of 4 min at 94 °C; 30 cycles of 45 s at 94 °C; 1 min at 72 °C; 1 cycle of 2 min at 72 °C	433	McClure et al. (2006)

### Sequencing of 16S rRNA genes

**Fig. 1:**
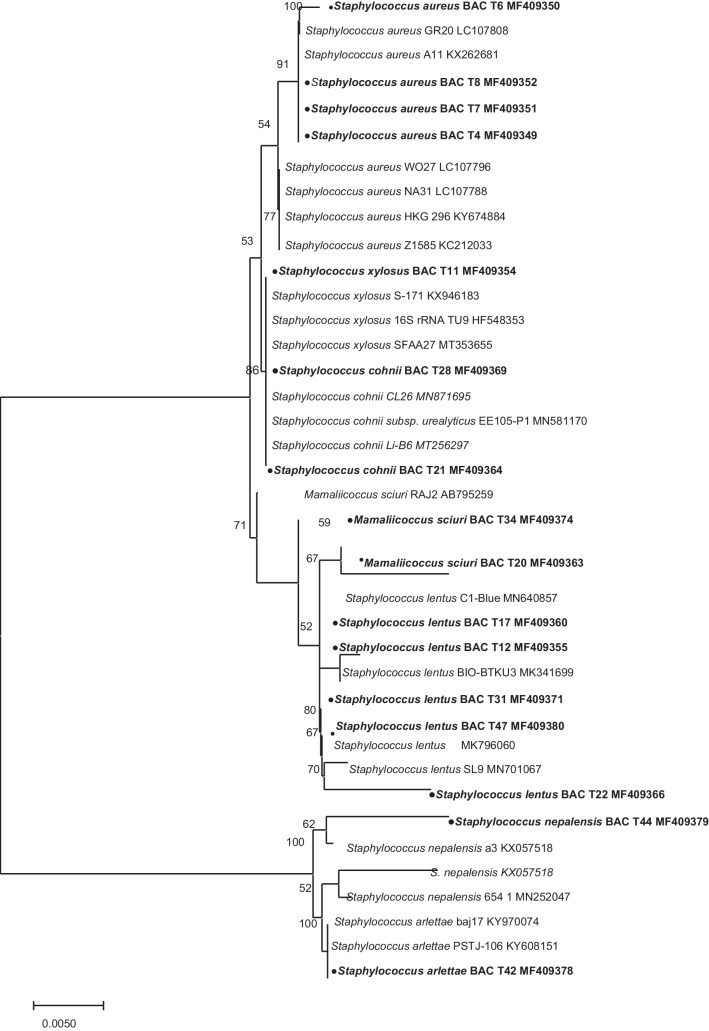
Unrooted neighbor-joining tree of *Staphylococcus* spp. isolated from a wastewater treatment plant in South Africa. Sequences obtained in this study are indicated as shaded circles. Accession numbers are indicated in bold. Neighbor-joining tree was constructed in MEGA (v. 6) using the Tamura-Nei substitution model replications. Bootstrap values below 50 are not shown

Purified PCR products were sequenced using the Big Dye terminator V. 3.1 cycle sequencing kit (Applied Biosystems, Warrington, UK) on a SeqStudio genetic analyzer and related software (Life Technologies, Holdings Pte Ltd, Singapore). Generated sequence electropherograms were inspected and then manually edited as described by Oladipo et al. (2018a). Edited sequences were aligned and compared against other sequences on the Basic Local Alignment Search Tool (BLAST) program alignment tool of the GenBank on the National Center for Biotechnology Information (NCBI) database (https://www.ncbi.nlm.nih.gov/). Phylogenetic sequence dendogram was constructed with closely related sequences obtained from GenBank by the neighbor-joining tree method using the Tamura–Nei substitution model in MEGA (Fig. 1). The partial 16S rRNA sequences obtained from this study are available in the GenBank with assigned accession numbers: MF409347–MF409381.

### PCR amplification of *mecA*, *nuc* and *luk-pvl* genes in staphylococcal species

To differentiate MRSA from other staphylococci the PCR amplification of the *mecA* gene (encoding for methicillin resistance), *nuc* gene and the *luk-pvl* gene that encode for virulence in staphylococcal species were conducted. Each of the PCR reaction contained 12.5 µL, 2 × PCR Master mix (Thermos Scientific Technologies, Waltham, MA, USA), 50 ng DNA template, 5 µM each of the primers (forward and reverse), and nuclease-free water added to a final volume of 25 µL. Detailed information on the primers and conditions used are presented in Table 1. To determine if the PCRs worked, electrophoresis of the amplicons were performed using a 1% w/v agarose gel and conditions described in Oladipo et al. (2018a). Previously known positive genes of *mecA*, *nuc*, and *pvl* and positive *Staphylococcus aureus* isolates were used as control strains.

### DNA amplification and sequencing of the protein A (*spa*)

For amplification of the *Staphylococcus* repeat region, a PCR was performed in a total volume of 50 μl containing cleaned DNA, 200 μM deoxynucleoside triphosphates (dATP, dCTP, dGTP, and dTTP), 10 pmol of each primer, 5 μl of tenfold concentrated PCR Buffer II (Applied Biosystems), MgCl_2_ 1.5 mM, and 1.25 U of AmpliTaq DNA polymerase (Applied Biosystems, Hitachi, Tokyo, Japan). Detailed information on the primers and conditions used are presented in Table 1.

Sequencing of the protein A gene (*spa*) was carried out using the Big Dye terminator V. 3.1 cycle sequencing kit (Applied Biosystems, Warrington, UK) on a 3130 Genetic analyzer (Applied Biosystems/Hitachi, Tokyo, Japan). The chromatograms obtained were analyzed with the Ridom *Staph* Type software version 1.4 (RidomGmbH, Sedanstr, Germany; http://spa.ridom.de/index.shtml). Spa types were deduced by the differences in number and sequence of spa repeats with the BURP algorithm (Ridom GmbH, Sedanstr, Germany) and the Ridom Spa Server database. Spa types with less than five or equal to 5 repeat units were excluded (Montanaro et al. 2016).

### Antimicrobial susceptibility testing

All isolated and identified staphylococcal species were subjected to antibiotic susceptibility testing of 13 antibiotics using the standard Kirby-Bauer’s disk diffusion technique (CLSI 2014). The specific antibiotics selected are beta-lactam antibiotics, a class of antibiotic that contain a beta-lactam ring in their molecular structures, that usually acts by inhibiting the synthesis of bacterial cell walls. This includes ampicillin, cloxacillin, amoxicillin, and methicillin. Others were macrolide-erythromycin, azithromycin, aminoglycoside gentamycin, carbapenems (imipenem), and glycopeptides (vancomycin). Methicillin was used to determine the antibiotic sensitivity of *Staphylococcus aureus* to other penicillin facing β-lactam resistance.

Antimicrobial resistance data were analyzed using the WHONET 2017 software V 5.6 (WHO; http://www.whonet.org/software.html). The multiple antibiotic resistance (MAR) index for the dominant isolates (*S. aureus*, *S. lentus*, and other staphylococcal species) at the influent and effluent compartments of the WWTP was calculated and interpreted according to Krumperman (1983) using the formula:

$$MAR\mathit\;index\mathit\;per\mathit\;compartment=\frac{\begin{array}{c}\mathrm{number}\;\mathrm{of}\;\mathrm{isolates}\;\mathrm{in}\;\mathrm a\;\mathrm{specific}\;\mathrm{sample}\\\mathrm{population}\;\mathrm{resistant}\;\mathrm{to}\;\mathrm{antibiotics}\end{array}}{\begin{array}{c}(\mathrm{number}\;\mathrm{of}\;\mathrm{antibiotics}\;\mathrm{tested})\times(\mathrm{total}\;\mathrm{number}\;\mathrm{of}\;\mathrm{organisms}\;\mathrm{in}\;\mathrm{sample})\\\end{array}}$$

*MAR index values > 0.2 indicate high risk source of contamination (Krumperman 1983).

### Statistical analyses

Statistical difference of MAR index of the staphylococcal species was done using one-way analysis of variance (ANOVA) at 5% level of significance using IBM SPSS Statistics 25 (IBM Corporation, Armonk, NY, USA). Multiple sequence alignment was performed using MUSCLE (Edgar 2004) integrated into Molecular Evolutionary Genetics Analysis (MEGA) V. 7.0 (http://www.megasoftware.net/; Kumar et al. 2016).

## Results

### Prevalence and distribution of staphylococcal strains from the wastewater treatment plant

**Fig. 2:**
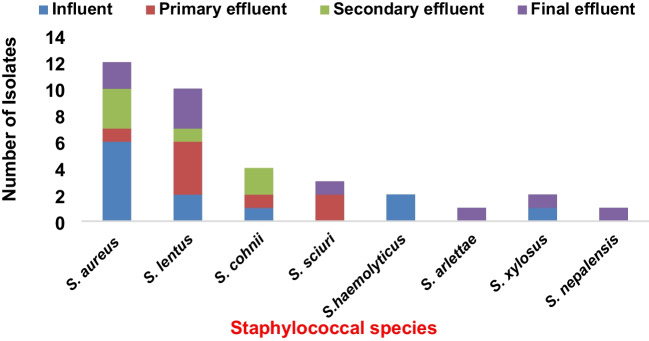
Distribution of staphylococcal species according to the sources of isolation

Thirty-five staphylococcal isolates belonging to eight (1 CoPS–*S. aureus* and 7 CoNS) species were identified. The most prevalent were *Staphylococcus aureus* (34.0%), *S. lentus* (29.0%), *S. cohnii* (11.0%), and *S. sciuri* (9.0%). Other isolates were *S. haemolyticus* and *S. xylosus* (6.0%) each and *S. nepalensis* and *S. arlettae* with 3% each*.* The phylogenic relatedness of the staphylococcus species alongside related GenBank sequences further confirmed these identities (Fig. 1). Twelve of the staphylococcal isolates {6 *S. aureus*, 2 *S. lentus*, 2 *S.haemolyticus*, 1 *S. cohnii*, and *S. xylosus* each} were from the influent, 8 of 35 (22.86%) comprising of *S. aureus* (1), *S. lentus* (4), *S. cohnii* (1), and *S. scuiri* (2) — {now reclassified as new genus, *Mammaliicoccus sciuri*} (Madhaiyan et al. 2020) from primary effluent. From the secondary effluent, 6 of 35 (17.14%) consisting of 3 *S. aureus*, 1 *S. lentus*, and 2 *S. cohnii* were identified, while 9 of 35 (25.71%) including *S. aureus* (2), *S. lentus* (3), *S. scuiri* (1), *S. arlettae* (1), *S. xylosus* (1), and *S. nepalensis* (1) were from the final effluent (Fig. 2). Furthermore, the distribution of the species across the four sampling points showed that *S. aureus* and *S. lentus* were isolated from all the sampling compartments. In addition, *S. arlettae* and *S. nepalensis* were exclusively isolated from the final effluent point while *S. xylosus* was isolated from the influent and the final effluent.

### Antibiotic resistance and susceptibility patterns of Staphylococcal species

**Fig. 3:**
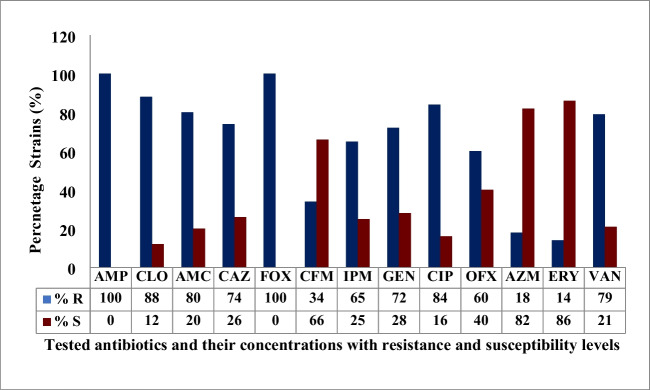
Antibiotic resistance profile of the *Staphylococcus aureus* strains isolated from the wastewater treatment plant

All thirty-five staphylococcal isolates from the wastewater treatment plant were subjected to 13 antibiotics at recommended concentrations for prove of resistance or susceptibility (Table 2). All the isolates were resistant to several of the 13 antibiotics tested. Resistance to the various antibiotics was in the following order: methicillin (91%), ampicillin (89%), ciprofloxacin (86%), amoxycillin (80%), ceftazidime (74%), and cloxacillin (71%). Other antibiotics are cefuroxime and azithromycin (43%), ofloxacin and vancomycin (40%), gentamycin (37%), imipenem (29%), and erythromycin (23%). About 70% (24 out of 35) of the isolates were resistant to at least 7 of the 13 antibiotics tested with *S. aureus*, *S. lentus*, and *S. scuiri* resistant to 10 of the 13 antibiotics (Table 2). It was observed that all the *S. aureus* strains isolated from the treatment plant regardless of their site of isolation were all resistant to methicillin and ampicillin (Table 2; Fig. 3). *Staphylococcus aureus* decreased from 50 to 17% as treatment progressed from influent to final effluent point in the WWTP. Furthermore, it was observed that 50% of *S. aureus* were resistant to imipenem and erythromycin.

**Table 2:** Identification, source, antibiotic resistance, and susceptibility pattern of staphylococcal species isolated from the wastewater treatment plant

S/N	Isolates	Accession number	Source of isolation	Antibiotics tested													Total	
				AMP	CLO	AMC	CAZ	FOX	CFM	IPM	GEN	CIP	OFX	AZM	ERY	VAN	R	S
1	*S. aureus*	MF409348	Influent	+	+	-	+	+	+	-	+	+	+	+	-	+	10	3
2	*S. aureus*	MF409349	Influent	+	+	+	+	+	+	-	+	+	+	-	-	+	10	3
3	*S. aureus*	MF409351	Influent	+	+	+	+	+	-	+	+	+	-	+	+	-	10	3
4	*S. aureus*	MF409356	Influent	-	+	+	+	+	+	+	+	+	+	+	-	-	10	3
5	*S. aureus*	MF409352	Influent	+	+	+	-	+	-	+	-	+	+	+	+	-	9	4
6	*S. cohnii*	MF409347	Influent	+	+	+	-	-	+	-	+	+	-	+	-	+	8	5
7	*S. lentus*	MF409357	Influent	+	+	+	+	+	+	-	-	-	+	+	-	-	8	5
8	*S. haemolyticus*	MF409358	Influent	+	+	+	+	+	+	+	-	+	-	-	-	-	8	5
9	*S. xylosus*	MF409354	Influent	+	+	+	+	-	-	-	-	+	+	-	+	-	7	6
10	*S. lentus*	MF409355	Influent	-	+	+	+	+	-	+	-	+	+	-	-	-	7	6
11	*S. aureus*	MF409350	Influent	+	+	+	+	+	-	-	-	-	-	+	+	-	6	7
12	*S. haemolyticus*	MF409353	Influent	+	+	-	-	-	-	-	+	+	-	-	-	-	4	9
13	*S. lentus*	MF409361	Primary effluent	+	+	-	+	+	-	+	+	+	+	+	-	+	10	3
14	*S. sciuri*	MF409363	Primary effluent	+	+	+	+	+	+	-	+	+	+	-	-	+	10	3
15	*S. aureus*	MF409359	Primary effluent	+	+	+	+	+	-	+	-	+	+	-	-	-	8	5
16	*S. lentus*	MF409360	Primary effluent	+	+	+	+	+	-	+	-	+	-	+	-	-	8	5
17	*S. sciuri*	MF409362	Primary effluent	+	+	-	+	+	-	-	+	+	+	+	-	+	8	5
18	*S. cohnii*	MF409364	Primary effluent	+	+	-	-	+	+	-	-	+	-	-	-	+	6	7
19	*S. lentus*	MF409365	Primary effluent	+	-	+	-	+	+	-	-	+	-	-	+	-	6	7
20	*S. lentus*	MF409366	Primary effluent	+	-	+	-	+	-	-	+	+	-	-	-	+	6	7
21	*S. aureus*	MF409367	Secondary effluent	+	-	+	+	+	-	+	-	+	+	-	-	+	8	5
22	*S. aureus*	MF409370	Secondary effluent	+	-	+	+	+	+	-	+	+	-	-	+	-	8	5
23	*S. cohnii*	MF409368	Secondary effluent	+	+	+	+	+	+	-	-	-	-	+	-	-	7	6
24	*S. lentus*	MF409371	Secondary effluent	-	+	+	+	+	-	-	-	+	-	+	-	-	6	7
25	*S. aureus*	MF409372	Secondary effluent	+	+	+	+	+	-	-	-	+	-	-	-	-	6	7
26	*S. cohnii*	MF409369	Secondary effluent	+	-	-	+	+	-	-	+	-	-	+	-	-	5	8
27	*S. aureus*	MF409381	Final effluent	+	+	+	+	+	+	-	-	+	+	-	-	+	9	4
28	*S. nepalensis*	MF409379	Final effluent	+	+	+	+	+	+	-	-	-	-	+	-	+	8	5
29	*S. sciuri*	MF409374	Final effluent	+	-	+	-	+	+	-	-	+	+	-	-	+	7	6
30	*S. lentus*	MF409375	Final effluent	+	+	+	-	+	+	-	-	+	-	+	-	-	7	6
31	*S. xylosus*	MF409376	Final effluent	-	-	+	+	+	-	+	+	+	-	-	+	-	7	6
32	*S. arlettae*	MF409378	Final effluent	+	-	+	+	+	-	-	-	+	-	-	+	+	7	6
33	*S. aureus*	MF409373	Final effluent	+	-	+	+	+	+	-	-	+	-	-	-	-	6	7
34	*S. lentus*	MF409380	Final effluent	+	+	-	+	+	-	-	-	+	-	-	-	+	6	7
35	*S. lentus*	MF409377	Final effluent	+	+	+	-	+	-	-	-	+	-	-	-	-	5	8
Total resistance obtained per antibiotic				**31**	**25**	**28**	**26**	**32**	**16**	**10**	**13**	**30**	**14**	**15**	**8**	**14**		

R indicates “resistant” and S “susceptible.” AMP (ampicillin) (10 μg), CLO (Cloxacillin) (5 μg), AMC (amoxycillin) (20 μg), CAZ (ceftazidime) (30 μg), FOX (methicillin) (30 μg), CFM (cefuroxime) (5 μg), IPM (imipenem) (10 μg), GEN (gentamycin) (10 μg), CIP (ciprofloxacin) (5 μg), OFX (ofloxacin) (5 μg), AZM (azithromycin) (15 μg), ERY (erythromycin) (15 μg), and VAN (vancomycin) (10 μg)

### Multiple antibiotic resistance (MAR) index and MAR phenotypes

The MAR index and phenotypes of the 2 dominant isolates (*S. aureus* and *S. lentus*) and other staphylococcal species comprising *S. arlettae*, *S. haemolyticus*, *S. sciuiri*, and *S. cohnii* are presented in Table 3. For *S. aureus* strains, the MAR index at the influent and effluent were 0.705 and 0.615, while for *S. lentus*, 0.577 and 0.692 were calculated. Furthermore, the MAR index of the other staphylococci — *S. arlettae*, *S. haemolyticus*, *S. sciuiri*, and *S. cohnii* (grouped as staphylococcus species) — were calculated as 0.519 for influents and 0.558 for effluents. However, there were no significant differences (*p* > 0.05) in the MAR index either within the sampling sites or among the different species. Also, the dominant phenotypes exhibited diverse patterns (Table 3). The MAR phenotype among *S. aureus* isolates from the influent showed diverse resistance to beta-lactam antibiotics and cephalosporins. The phenotypes — AMP-CLO-AMC-CAZ-FOX and AMP-CLO-AMC-CAZ-FOX-CFM — were dominant in *S. aureus* (16.7%), *S. lentus* (50.0%), and other staphylococci (33.3%) from the influent chamber of the WWTP. However, at the effluent chamber, the MAR phenotypes, AMP-CLO-AMC-CAZ-FOX-CFM-CIP-ERY-VAN (*S. aureus*), AMP-CLO-AMC-CAZ-FOX-CIP-VAN (*S. lentus*), and AMP-CLO-AMC-CAZ-FOX-CFM-AZM (*Staph* spp.), were observed in 50%, 25%, and 33.3% of the isolates, respectively.

**Table 3:** MAR phenotypes and MAR index among the staphylococcal species from the WWTP

Isolates	Source(s)	MAR phenotype	No observed	%	Group MAR index
*S. aureus*	Influent *n* = 6	AMP-CLO-CAZ-FOX-CFM-GEN-CIP-OFX-AZM-VAN AMP-CLO-AMC-CAZ-FOX-CFM-GEN-CIP-OFX-VAN AMP-CLO-AMC-CAZ-FOX -AZM-ERY AMP-CLO-AMC-CAZ-FOX-IMP–GEN-CIP-AZM-ERY AMP-CLO-AMC-FOX-IPM-CIP-OFX-AZM-ERY CLO-AMC-CAZ-FOX-CFM-IPM-GEN-CIP-OFX-AZM	1 1 1 1 1 1	16.7 16.7 16.7 16.7 16.7 16.7	0.705
	Effluent *n* = 2	AMP-AMC-CAZ-FOX-CFM-CIP-ERY-VAN AMP-CLO-AMC-CAZ-FOX-CFM-CIP-ERY-VAN	1 1	50.0 50.0	0.615
*S. lentus*	Influent *n* = 2	CLO-AMC-CAZ-FOX-IPM-CIP-OFX-AZM AMP-CLO-AMC-CAZ-FOX-CFM-OFX-AZM	1 1	50.0 50.0	0.577
	Effluent *n* = 4	AMP-CLO-AMC-FOX-CFM-CIP-AZM AMP-CLO-AMC-CAZ-FOX-CIP-VAN AMP-CLO-AMC-FOX-CIP AMP-AMC-CLO-AMC-CAZ-CIP-OFX-ERY	1 1 1 1	25.0 25.0 25.0 25.0	0.692
Other *Staph. species*	Influent *n* = 3	AMP-CLO-GEN-CIP AMP-CLO-AMC-CAZ-FOX-IPM-CIP AMP-AMC-CLO-AMC-CAZ-CIP-OFX-ERY	1 1 1	33.3 33.3 33.3	0.519
	Effluent *n* = 3	AMP-AMC-CAZ-FOX-CIP-ERY-VAN AMP-CLO-AMC-CAZ-FOX-CFM-AZM AMC-CAZ-FOX-IPM-GEN-CIP	1 1 1	33.3 33.3 33.3	0.558

### Detection of resistance and virulence genes and in staphylococcal species

Seventy-seven percent of the isolates (27 of 35) were *mecA* positive. Among the 12 MRSA isolates, 11 were *mecA* positive. Other staphylococci that tested positive for *mecA* were *S. lentus*, *S. scuiri *(*Mammaliicoccus sciuri*), *S. cohnii*, *S haemolyticus*, and *S. xylosus*. This study revealed a higher number of isolates being recovered in the final effluent; however, 75% of these isolates did not carry the *mecA* resistance gene. Two (5.8%) *S. aureus* isolates were also positive for the *luk-pvl* gene, while the *nuc* gene was detected in all 12 *S. aureus* isolates (Table 4).

**Table 4:** Detection of *mecA*, *pvl*, and *nuc* genes and *Spa* types of *S. aureus* isolates from the wastewater treatment plant

Source(s)	Isolates (*n* = 12)	*mecA*	*pvl*	*nuc*	Spa type	Spa repeats	Sequence type (ST)
Influent	*S. aureus*	+	-	+	*t061	09–02-16–13-34–17-34–16-34	ND
Influent	*S. aureus*	+	-	+	UNK	23–21-17–34-12–23-02–12-23	ND
Influent	*S. aureus*	+	-	+	*t6578	26–23-13–21-17–34-33–34	**ST-398**
Influent	*S. aureus*	+	+	+	UNK	13–12-16–34-33–13	ND
Influent	*S. aureus*	+	-	+	*t091	07–23-21–17-34–12-23–2-12–23	ND
Influent	*S. aureus*	+	-	+	UNK	34–34-34–34-34–17-34–16-13	ND
P. effluent	*S. aureus*	+	-	+	*t447	26–23-34–17-20–17-12–17-16	ND
S. effluent	*S. aureus*	+	-	+	*t7835	7–82-21–17-34–34-16–34-33–13	**ST-15**
S. effluent	*S. aureus*	+	-	+	UNK	34–34-12–12-23–2-12–23	ND
S. effluent	*S. aureus*	+	+	+	*t657	23–13-21–17-34–33-34	**ST-772**
F. Effluent	*S. aureus*	+	-	+	*t091	07–23-21–17-34–12-23–2-12–23	**ST-7**
F. Effluent	*S. aureus*	-	-	+	*t5126	26–23-12–34-34–12-12–23-12–23	ND

The *Spa* types in this study are asterisked. In some cases, the clonal complex was assumed according to the spa-type in this case it is bolded, UNK denotes “unknown” while means “not done.” The isolate sources are indicated as: P. effluent (primary effluent), S. effluent (secondary effluent), and F. effluent (final effluent), while the sequence type denoted as ND means “not detected”

### Detection of protein A (*spa*) types in Staphylococcus aureus strains

Seven different *spa* types were detected from the confirmed *S*. *aureus* strains recovered from the WWTP in this study (Table 4). *Spa* types t061, t6578, and t091 were detected at the influent, t447 from primary effluent, t7835 from secondary effluents, and *spa* types t091 and t5126 final effluent. The most frequent *spa* type t091 (16.7%) was observed from the influent and secondary effluent.

## Discussion

Several researchers in South Africa have investigated links between WWTP effluents and receiving waters. These have focused on physico-chemical properties (Agoro et al. 2018; Salvador-Oke et al. 2018) or microbial parameters (microorganisms) such as *Vibrio* (Okoh & Igbinosa 2010) and *Aeromonas* species (Igbinosa & Okoh 2012; Coetzee et al. 2017; Mann et al. 2019). The present study was designed to assess the prevalence of staphylococcal species (*S. aureus* — CoPs and CoNS), their antibiotic resistance patterns, and detection of resistance and virulence genes and *Spa* types in the recovered staphylococcal species from a WWTP in South Africa.

In the present study, eight staphylococcal species (1 CoPS and 7 CoNS) were isolated and identified across the 4 sampling points of a wastewater treatment plant in South Africa. These are *Staphylococcus aureus*, *S. arlettae*, *S. cohnii*, *S. haemolyticus*, *S. lentus*, *S. nepalensis*, *S. sciuri*, and *S. xylosus*. The *Staphylococcus* spp. demonstrated multidrug resistance (MDR), high MAR index (> 0.2), various MAR phenotypes, detection of *mecA* resistance gene, and the *nuc* and *luk-pvl* virulence gene and *Spa* types confirmed in *S. aureus* strains.

This study confirmed the presence of staphylococci in the final effluent after chlorination. Due to its simple management, low cost, and high efficiency in eliminating microorganisms in wastewater treatment plants, chlorination purification method at the final/tertiary effluent phase was considered an effective disinfection method (Wang et al. 2020; Collivignarelli et al. 2021). However, in recent times, chlorination has proved to transmit antibiotic resistance genes (ARGs) in treated wastewaters (Ghernaout & Elboughdiri 2020; Collivignarelli et al. 2021). Hence, this process may not lethally affect microorganisms including staphylococci in wastewaters (Liu et al. 2018; Collivignarelli et al. 2021). This may explain the detection of antibiotic-resistant staphylococcal species after chlorination in the wastewater treatment plant in this study.

WHO (2017b) had earlier reported chlorine resistance of staphylococci species. Previous studies (Huang et al. 2011; Shi et al. 2013; Mao et al. 2015) reported the presence of antibiotic-resistant bacteria that revealed resistance to chlorination. In addition, Gómez et al. (2016) confirmed the detection of multi-drug resistant staphylococci (*S. aureus*, *S. lentus*, *S. cohnii*, *S. scuiri *(*Mammaliicoccus sciuri*), and *S. haemolyticus*) in urban wastewater treatment plant in Spain at the final effluent phase. Similarly, Goldstein et al. (2012) and Maimon et al. (2014) recorded the occurrence of *S. aureus* and methicillin-resistant *S. aureus* (MRSA) in treated wastewater effluents from greywater, intended for reuse. Hence, in order to eliminate the presence of microorganisms such as staphylococci species from treated wastewaters, the use of ultraviolet (UV) radiation has been suggested as a promising and more effective treatment technology (Collivignarelli et al. 2021).

All *S. aureus* isolates in the present study were resistant to ampicillin and methicillin. This appears to be a constant observation amongst previous studies. Thapaliya et al. (2017) also recorded the prevalence of *S. aureus* and their antibiotic-resistance in wastewater treatment plant sites. Their study investigated the prevalence and molecular characteristics of *S. aureus* and MRSA in freshwater recreational beaches sand and water samples collected from 10 beaches in Northeast Ohio, USA. Results from their study revealed overall prevalence of *S. aureus* (22.8%) and *PVL* genes (21.4%), with 27 different *spa* types identified. In addition, 34.3% of the isolates showed oxacillin resistance while, all the isolates showed 100% resistance to penicillin. However, our present study revealed a higher prevalence of *S. aureus* (34.3%) with a prevalence of 5.71% for *PVL* genes and 7 *spa* types being identified among *S. aureus* isolates.

Results of Thompson et al. (2012) and Porrero et al. (2016) showed that 96% and 83% MRSA isolates, respectively, from urban effluents were resistant to ampicillin. The presence of MRSA and MSSA in river water and urban effluents was studied to analyze the *S. aureus* population and determine the genetic diversity. From their study, MRSA population in urban effluents and river water was 67.6% and 82.4%, while spa type t067 was the predominant MRSA genotype detected. This differs from our study in that we only recorded an MRSA prevalence of 35%, in a WWTP with spa type t091 being dominant. Said et al. (2017) reported that most of the *S. aureus* in their study showed resistance to penicillin, while Goldstein et al. (2012) also demonstrated that 93% of MRSA isolates recovered from wastewaters in the USA were multidrug resistant. The study by Goldstein et al. (2012) examined the occurrence of MRSA and methicillin-susceptible *S. aureus* (MSSA) at US wastewater treatment plants. The study and findings were similar to this study since the presence of MRSA in a WWTP was investigated. Results from their study also showed 10 of 12 (83%) influent samples being MRSA-positive, while one of 12 (8%) effluent samples was MRSA-positive.

In the present study, it was shown that the *mecA* resistance gene was detected in 11 of the 12 *S. aureus* strains recovered from the WWTP sampled. This finding is corroborated by several previous studies (Wan & Chou 2014; Boopathy 2017). While Boopathy (2017) established the presence of methicillin-resistant *Staphylococcus aureus* (MRSA) in a rural sewage treatment plant, Wan and Chou (2014) examined the spreading of *β*-lactam resistance gene (*mecA*) and methicillin-resistant *Staphylococcus aureus* through municipal and swine slaughterhouse wastewaters.

The *nuc* gene was detected in all the *S. aureus* strains isolated from this study. However, the *pvl* virulence gene was detected in very few isolates. A similar study of clinical isolates (von Eiff et al. 2004) examined the prevalence of genes encoding for members of the staphylococcal leukotoxin family of *Staphylococcus aureus*. Their findings revealed 0.9 to 1.4% detection of *pvl* virulence gene.

The occurrence of CoNS in WWTPs has also been well documented. Faria et al. (2009) reported the survival of CoNS in treated effluents. On the other hand, Čuvalova et al. (2015) demonstrated that CoNS also occurred in drinking water. Of the 7 CoNS species identified in this study, Gómez et al. (2016) and Said et al. (2017) detected 5 species in their studies that focused on wastewater samples. These species included *S. cohnii*, *S. haemolyticus*, *S. lentus*, *S. scuiri *(*Mammaliicoccus sciuri*), and *S. xylosus* from wastewater samples. Borjesson et al. (2009) recovered *S. cohnii*, *S. haemolyticus S. lentus*, and *S. sciuri* from a municipal wastewater treatment plant. Antibiotics resistance by CoNS had also been documented. This was the case in the present study and several previous studies. Said et al. (2017) reported that CoNS isolated from wastewaters in Tunisia were resistant to several classes of antibiotics including beta-lactam antibiotics. Previously, Schwartz et al. (2003) had reported the occurrence of methicillin-resistant CoNS from wastewater environments. The detection of *mecA* resistance gene in CoNS has also been reported in recreational waters, community, and hospital wastewaters (Börjesson et al. 2009; Fogarty et al. 2015) and in other surface waters (Seyedmonir et al. 2015). Finding CoNS strains in the wastewater from the present North West Province of South Africa is thus not extraordinary.

The multiple antibiotic resistance (MAR) index of all 35 (100%) *Staphylococcus* spp. in our study exceeded the 0.2 value associated with highly antibiotic resistant strains. In a previous study (Oladipo et al. 2019), very high MAR index (> 0.2) were also recorded for *S. aureus* isolates from clinical and environmental sources. Multiple antibiotic resistance in bacteria is most commonly associated with the presence of plasmids which contain one or more resistance genes, each encoding a single antibiotic resistance phenotype. MAR index values greater than 0.2 indicate high risk source of contamination where antibiotics are often used. Our findings in the present study indicate that the presence of staphylococcal species exhibiting antibiotic resistance and harboring environmentally relevant virulence genes are of particular concern due to the possible link of community acquired MRSA and wastewater recycling for domestic, agricultural, and industrial use. The frequencies of resistance of *S. aureus* to beta-lactams antibiotics (AMP-CLO-AMC-CAZ-FOX) were high at all our sampling sites (influent, primary, and secondary effluents and final effluent). Several studies have shown *S. aureus* resistance to antibiotics such as penicillin, amoxicillin, and/or ampicillin have been isolated from both treated and untreated wastewater (Sahlstrom et al. 2004; Feng 2008). In a previous study carried out in the USA, increase percentages of Ery-, Amp-, and Pen- -resistant were also reported among staphylococcal species isolated from a WWTP (Goldstein et al. 2012).

Seven distinct *spa* types were identified in this study with t091 being the most prevalent. Finding such a variety of *spa* types is a potential indication of diverse sources of isolation and that these could be from different geographical locations. This was also observed in a study in Nigeria (O'Malley et al. 2015; Ayeni et al. 2018) where *spa* type t091 was confirmed in nasal samples of clinical and poultry sources. In addition, Ilczyszyn et al. (2016) reported occurrence of *spa* type t091 amongst 5-year-old and younger patients in Poland. However, no data in searched databases could be found for a South African study on MRSA that had documented *spa* type t091 being associated with wastewaters.

The *spa* type t7835 had been associated with MRSA from clinical isolates in Nigeria (Kolawole et al. 2013), while *spa* type t447 had been reported in Netherlands and Spain. Also, *spa* type t6578 had been identified among swine (LA-MRSA) in Spain and the USA as ST398 (CC398), and subsequently detected in several companion and food-chain animals and humans (de Boer 2009). According to Smith et al. (2009), ST398 (CC398) has been well reported as a cause of livestock-associated (LA)-MRSA in Europe, while in Australia (Price et al. 2012) and the Americas (Grema et al. 2015), ST398 had been confirmed as a cause of LA-MRSA. *Spa* type t5126 had been identified in MRSA strains in Spain, USA, Germany, and France (https://spa.ridom.de/spa-t5126.shtml ), while s*pa* type t061 had been associated with MRSA in the UK, Germany, and USA (von Eiff et al. 2008). Notably, *spa* type t657, sequence type (ST)772, was reported in this study among the *pvl* positive strains. This sequence type had been linked to community outbreak of CA-MRSA infections in some parts of the world, e.g., India (D'Souza et al. 2010) and Ireland (Edmundson et al. 2011).

In this study, *S. aureus* constituted 34% of the total recovered staphylococcal species which decreased as treatment progressed from influent to final effluent point. Similarly, studies conducted in Sweden, Spain, and the USA, respectively, reported 50–55% prevalence of *S. aureus* in WWTPs with decreased prevalence as treatment progressed (Börjesson et al. 2009; Goldstein et al. 2012; Gómez et al. 2016). In this study, the higher number of isolates from the final effluent which did not carry the *mecA* resistance gene could potentially be similar to the findings of Mao et al. (2015) that also reported a reduction of antibiotics resistance genes (ARGs) and *mecA* gene from raw influent point to the effluent.

In this study, three CoNS-*S. nepalensis*, *S. arlettae*, and *S. cohnii* from the WWTP which have not been widely reported were detected. Of these, *S. cohnii* carried the *mecA* resistance gene. Nováková et al. (2006) had earlier isolated *S. nepalensis* from human urine, *S. arlettae* from textile effluents (Elisangela et al. 2008), while *S. cohnii* had been recovered from wastewater samples (Börjesson et al. 2009; Gómez et al. 2016; Said et al. 2017). *Staphylococcus cohnii* is known for its associations with nosocomial infections (Chen et al. 2015) and had also been confirmed in infections in animals (Sousa et al. 2014).

Previously, CoNS had been regarded as non-pathogenic since most of these species are established by association between humans and animals (Otto 2010). However, their antibiotic resistance traits, possession of resistance, and virulence genes reveal evidence that these species could have detrimental human health outcomes and should therefore be studied more closely.

## Conclusions

The present study demonstrates that environmental waters that receive WWTP effluent could be contaminated with MRSA and other potential pathogenic staphylococci. These findings indicate the possibility of treated wastewaters being a source for the dissemination of staphylococcal species, their resistance, and virulence genes to the environment which could have detrimental health impacts on the downstream users and consumers. The detection of a large proportion of MAR isolates in the present study is a cause for concern as this could pose health risks to humans and animals via resistant genetic elements that could be transferred from these isolates to other bacteria also of clinical importance. Therefore, a more effective treatment plan or treatment modification procedures of wastewaters such as ultraviolet (UV) radiation may therefore be crucial, especially if the water is to be reused. The findings of the present study are aspects that the managers of wastewater treatment plants, policy formulators, down-stream users, etc., should consider.

## Data Availability

The datasets used and/or analyzed during the current study are available from the corresponding author on reasonable request.

## Declarations

### Disclaimer

The views expressed are those of the authors and not of the funders.
